# Effect of Na versus Ca Sulfate Salts on the Hydration of Calcium Sulfoaluminate Clinker

**DOI:** 10.3390/molecules28227651

**Published:** 2023-11-18

**Authors:** Pilar Padilla-Encinas, Angel Palomo, Ana Fernández-Jiménez

**Affiliations:** Eduardo Torroja Institute for Construction Science, National Research Council (iETcc-CSIC), 28033 Madrid, Spain; maria.padilla@uam.es (P.P.-E.); palomo@ietcc.csic.es (A.P.)

**Keywords:** calcium sulfoaluminate clinker, alkaline salts, microstructure, calorimetry

## Abstract

This paper examined how the amount (5% or 20%) and type (CaSO_4_ or Na_2_SO_4_) of sulphate salt affect the hydration of calcium sulphoaluminate clinker (KCSA). The mechanical behavior of the pastes was determined at 1, 3, 28, and 90 days, the heat flow and total heat were measured with isothermal conduction calorimetry, and the reaction products were characterized using X-ray diffraction (XRD), differential thermal analysis/thermogravimetry (DTA/TG) and scanning electron microscopy (SEM). The results obtained indicated that both the amount of sulphate salt (5% or 20%) and its type (CaSO_4_ or Na_2_SO_4_) affect the hydration kinetics, type of reaction products formed, and development of mechanical strength. The incorporation of CaSO_4_ has a positive effect on the development of the mechanical strength of KCSA. However, Na_2_SO_4_ can also produce adverse side effects at older ages. The presence of Na_2_SO_4_ increases pH values, which partly destabilizes the ettringite formed, thereby favoring carbonation and thenardite precipitation, which can cause the specimens to crack and break.

## 1. Introduction

The presence of a high alkali content during KCSA hydration has a negative effect on the development of mechanical strength. Conversely, a relatively low alkali content (0.1, 1 M) can have a positive effect. This paper investigates how the presence of solid chemical alkali activators (Na_2_SO_4_ type salts) affects KCSA hydration. The interest in knowing how this salt affects KCSA hydration stems from the possibility of producing hybrid alkaline cements (HACs) [1,2,3], in which these types of salts are used to increase the reactivity of the mineral additions (fly ash, slag) [4,5,6].

During the production of Portland-cement-based HACs, this neutral salt (Na_2_SO_4_) interacts with the Ca(OH)_2_ (produced by hydration of the Portland cement) to generate alkalinity in situ (see Equation (1)), which increases the reactivity of the mineral additions.

Ca(OH)_2_ + Na_2_SO_4_ + 2 H_2_O → CaSO_4_ · 2 H_2_O (s) + 2 Na^+^ OH^−^ (aq) (1)

In the hydration of KCSA or calcium sulfoaluminate (CSA), the formation of Ca(OH)_2_ is not favored, so we assume that this reaction does not take place. Therefore, it is important to know how the presence of Na_2_SO_4_ can affect KCSA hydration.

Many papers [7,8,9,10] in the literature have studied the effect of calcium sulfate on KCSA hydration. The presence of calcium sulfate (anhydrite or gypsum) leads to the formation of ettringite and aluminum hydroxide. Thus, it is estimated that 15–25% by mass is the appropriate amount of gypsum and/or anhydrite to be added to calcium sulfoaluminate clinker to optimize the setting time, development of mechanical strength and volume stabilization and to obtain a CSA.

In the hydration process of KCSA with calcium sulfate, Jansen et al. [11] distinguished the formation of two different types of ettringite: (i) first-generation ettringite, together with other compounds such as CAH_10_, amorphous AH_3_ and monosulfate, which corresponded to the first peak and induction period in the heat flow curves of isothermal conduction calorimetry; and (ii) second-generation ettringite, together with a microcystalline AH_3_, which corresponded to the main peak in the heat flow curves of isothermal conduction calorimetry. The presence of different shoulders in the heat flow curves was attributed by the authors to the recrystallization processes of microcrystalline AH_3_ and the final adjustment of the water content of the aluminum hydroxide towards AH_3_.

However, very few studies have investigated the effect of Na_2_SO_4_ on the hydration of KCSA or CSA. Sánchez-Herrero et al. [12] analyzed the effect of adding 4% Na_2_SO_4_ and 8% Na_2_SO_4_ on the hydration of C_3_A. They observed that the addition Na_2_SO_4_ favored the precipitation of the U-phase (C_4_A$\bar{s}$NH_16_), which is morphologically similar to calcium monosulfoaluminate. The same authors [13] studied the effect of 4% Na_2_SO_4_ on the hydration of synthetic ye’elimite. They observed the formation of the U-phase, which slightly favors the development of mechanical strength since the U-phase has the capacity to densify the material. Regarding the effect of these salts on the hydration of CSA, Palou et al. [14] observed that the hydration of CSA is faster in the presence of 1, 2, and 3% Na_2_SO_4_ than with water. This behavior is associated with the formation of a primary ettringite, probably on the surface of the ye’elimite. Durlo et al. [15] studied the effect of 5% Na_2_SO_4_ on the hydration of CSA cement. They observed two opposite effects: in the short term, the hydration of ye’elimite is accelerated, while in the long term, there is reprecipitation of thenardite, precipitation of gypsum and carbonation of the ettringite.

To determine the effect of Na_2_SO_4_ on the hydration of KCSA, two doses of this salt (5% and 20%) have been selected for this study. The first dose (5%) is the dosage normally used in Portland-cement-based HACs [16,17,18] and the second (20%) is chosen to enable comparison with the CaSO_4_ dosage used in CSAs [9,10]. CaSO_4_ is used at the same dosage (5% and 20%) to provide a reference, and some of the tests also included commercial CSA cement as a control material.

## 2. Results

### 2.1. Isothermal Conduction Calorimetry

**Figure 1:**
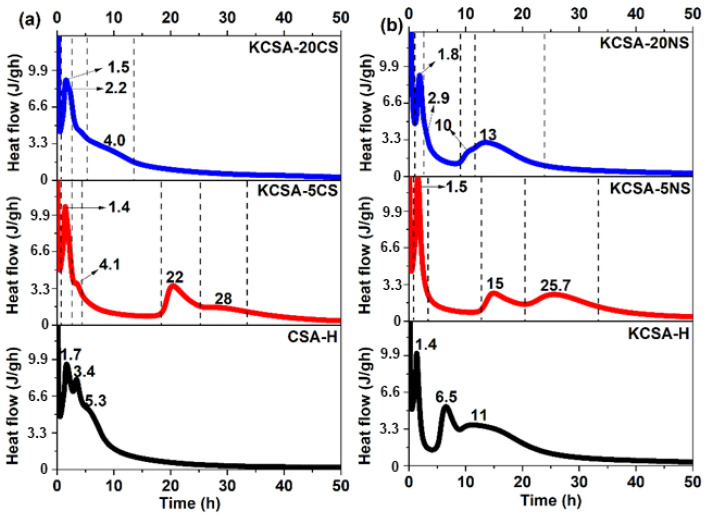
(**a**) Heat release rate of the pastes containing 5% and 20% CaSO_4_; (**b**) heat release rate of the pastes containing 5% and 20% Na_2_SO_4_.

The hydration kinetics of the calcium sulfoaluminate clinker were analyzed using isothermal conduction calorimetry in the presence of 5% and 20% Na_2_SO_4_ and CaSO_4_ (reference). This section also includes the hydration of a commercial CSA that should be considered a control material. The liquid (water)/solid ratio used in all cases was 0.5. The heat release rate results are shown in Figure 1.

The heat release rate curve for the water-hydrated KCSA paste (KCSA-H) shows four peaks, occurring, respectively, at 30 min, 1.4 h, 6.5 h, and 11 h (see Figure 1). The curve for the water-hydrated cement (CSA-H) also shows four peaks [15]. The first of these, which occurs at 30 min, is common to all the pastes. After that, two peaks and one shoulder are formed, reaching their highest values at 1.7 h, 3.4 h, and 5.3 h, respectively. Comparison of the two pastes (KCSA-H and CSA-H) shows that cement hydration generates remarkable heat release for approximately 20 h; during KCSA hydration, the heat release continues for around 30 h. While no induction period is evident in the cement (CSA-H), the clinker paste (KCSA-H) presents an induction period of 3 h.

When 5% CaSO_4_ is added to the KCSA (KCSA-5CS), five peaks are formed (Figure 1a). The first of these occurs at 30 min, followed by two peaks at 1.4 h and 4.1 h. A 13 h induction period is followed by one peak (22 h) and one shoulder (28 h). Increasing the calcium sulfate concentration to 20% (KCSA-20CS) results in three peaks and one shoulder. The first is common to all the previous pastes and is followed by two peaks that reach their maximum values at 1.5 h and 2.2 h, respectively. After that, one shoulder is formed at 4 h.

When comparing the heat release rate curves for the four pastes (CSA-H, KCSA-H, KCSA-5CS, KCSA-20CS), the main difference lies in the presence of an induction period before the third and fourth peaks in the pastes that have either zero calcium sulfate (KCSA-H) or a low calcium sulfate content (KCSA-5CS). However, all four pastes have a first peak that appears at around 30 min, followed by a second peak that also appears at similar times in all of them (between 1.4 and 1.7 h). These results corroborate those obtained by other authors [9,10], who indicate that the presence of CaSO_4_ content close to the stoichiometric ratio (20–22%) accelerates the hydration reaction of the KCSA.

When sodium sulfate is added instead of calcium sulfate, four signals are obtained for the KCSA-5NS paste, and five are obtained for the KCSA-20NS paste (Figure 1b). The first two peaks emerge at similar times to those in the pastes hydrated with water (after 30 min and between 1.4 and 1.8 h). In the paste containing 20% Na_2_SO_4_ (KCSA-20NS), the peak formed at 1.8 h is followed by a small shoulder at 2.9 h. The hydration process of the pastes containing the salt is then slowed by a long induction period, which is greatest in the KCSA-5NS paste. The last peaks appear at 15 h and 25.7 h in the KCSA-5NS and at 10 h and 13 h in the KCSA-20NS.

These results show that the sulfate type and concentration significantly affect the induction period and the third and fourth peaks of the heat release rate curves. Relatively low sulfate salt concentrations delay the third and fourth signals while high concentrations accelerate them. The first and second peaks, however, which, according to the literature [11,19], are associated with the formation of a primary ettringite, do not appear to be seriously affected.

**Figure 2:**
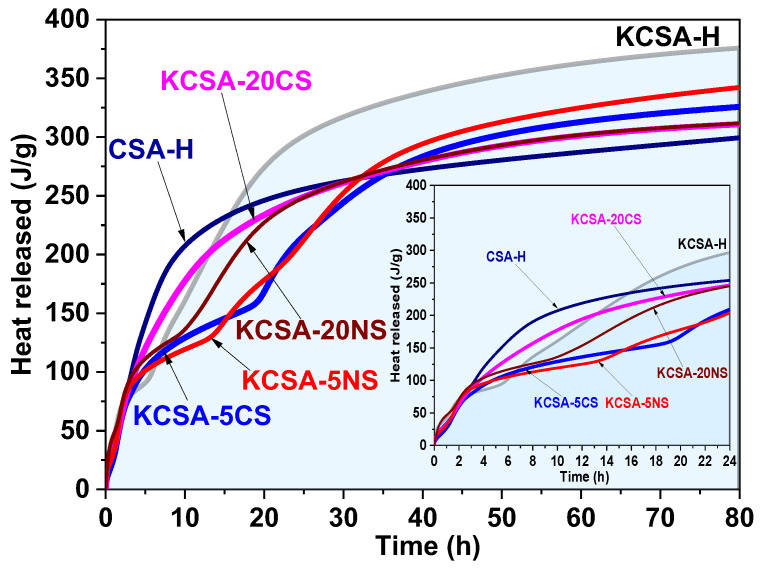
Total heat of the reference samples (KCSA-H and CSA-H) and of the pastes containing 5% and 20% CaSO_4_ and Na_2_SO_4_.

Figure 2 shows the total heat as a function of time. It demonstrates that for the first two hours, all the systems share very similar curves. Over longer time periods, the water-hydrated clinker with no added sulfates (KCSA-H) releases the most total heat. The KCSA-5CS and KCSA-5NS pastes release the least initial heat due to their delayed reaction rate, but at advanced ages, the heat released, although less than that released by the KCSA-H, is higher than that released by the pastes with a 20% sulfate content (KCSA-20CS and KCSA-20NS). The samples with the greatest calcium sulfate contents (CSA-H and KCSA-20CS) release the most heat initially, which is consistent with the acceleration of the reaction process observed in Figure 1. Although the KCSA-20NS paste initially releases slightly less heat than the other pastes, it behaves similarly to them at advanced ages. The lower amounts of heat released by these samples (CSA-H, KCSA-20CS, and KCSA-20NS) at advanced ages may be partly due to a dilution effect.

### 2.2. Mechanical Behavior

**Figure 3:**
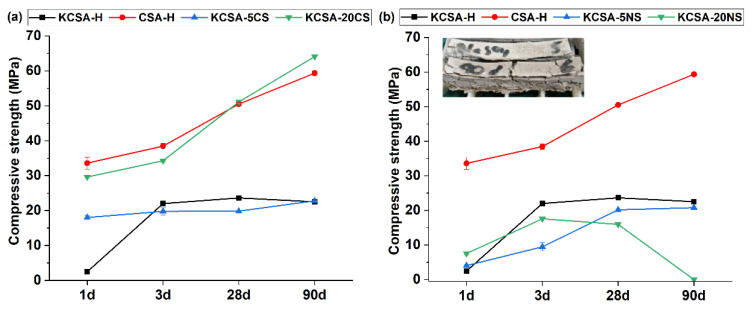
Compressive strength of the pastes containing 5% and 20% salt concentrations; (**a**) CaSO_4_; (**b**) Na_2_SO_4_.

Figure 3 shows the compressive strength values for the pastes at 1, 3, 28, and 90 days. As can be seen, the KCSA-5CS paste presents strength values close to those obtained by the KCSA-H, while the KCSA-20CS paste presents higher strength values at all ages, similar to those obtained by the cement (CSA-H).

The presence of sodium sulfate (Figure 3b) results in low strength values at all ages in the study, with the KCSA-20NS paste eventually fracturing at 90 days. Figure 3 includes a photograph of the cracked paste specimen at 90 days for which, due to the fracture, it was not possible to determine the mechanical strength. Similar results were obtained in another study [20], in which KCSA clinker was hydrated with an 8 M NaOH solution, and also in the papers published by Tambara et al. [15] on the hydration of a CSA with Na_2_SO_4_, and by Sánchez et al. [21] on the hydration of ye’elimite with 8 M NaOH.

Cement paste is a chaotic system with a very complex structure in which, in addition to the size and volume of pores, there are geometric characteristics that are difficult to quantify, irregular pore shapes and disordered spatial distributions of pores. The structure of the pores can be predicted indirectly through computational models, applying the concepts of fractal methodology [22] or thermodynamics. It can also be measured directly through experimental techniques such as nuclear magnetic resonance, nitrogen adsorption/desorption and mercury intrusion porosimetry (MIP). In this work, the latter method has been used. Although with this technique there are parameters that are not taken into account, such as the tortuosity of the system, it is considered that the information obtained may be sufficient for the desired objective.

**Figure 4:**
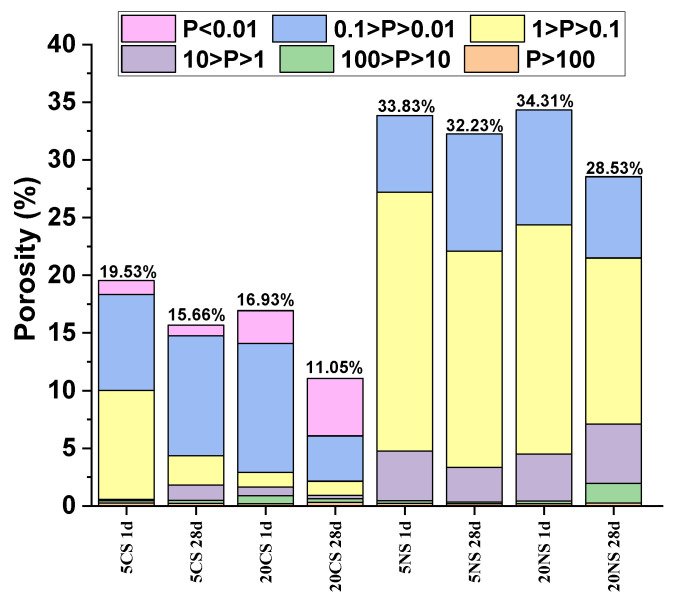
Porosimetry results at 1 and 28 days for the hydrated pastes containing 5% and 20% CaSO_4_ and Na_2_SO_4_.

Figure 4 shows the porosity values and pore size distribution of the pastes containing 5% and 20% CaSO_4_ and Na_2_SO_4_ at 1 and 28 days of hydration. The first observation is that, in every case, the porosity decreases with an increase in the hydration time. The figure also shows that the pastes containing CaSO_4_ clearly present lower porosity values and pore sizes than those obtained with Na_2_SO_4_. Based on the pore diameter in a hardened cement paste, pores can be classified as follows: *gel pores* (<0.01 μm), *small capillary pores* (0.01–0.1 μm), *large capillary pores* (0.1–1 μm), and *air pores* (>1 μm) [23]. The number of *capillary pores* depends on the water/binder (w/b) ratio and the degree of reaction of the paste, which tends to decrease as the degree of hydration increases. The pastes containing calcium sulfate generally have a higher percentage of *small capillary pores* (0.01–0.1 μm), while *large capillary pores* (0.1–1 μm) predominate in pastes containing sodium sulfate.

### 2.3. Mineralogical Characterization: Continuous X-ray Diffraction (XRD)

Figure S1 shows the continuous XRD (every 30 min) diffractograms obtained from the hydrated pastes (KCSA-5NS, KCSA-5CS, KCSA-20NS, KCSA-20CS) in the first 24 h. Different colors are used in the diffractograms depending on the time intervals established for each signal obtained during calorimetry (Figure 1).

The reduction in the intensity of the peaks associated with the ye’elimite is greater in the calcium sulfate pastes (KCSA-5CS and KCSA-20CS (Figure S1a,b) than in the sodium sulfate pastes (Figure S1c,d). The intensity of the peaks associated with the ettringite, meanwhile, is greater.

As regards the sulfate content, greater ye’elimite consumption appears to occur in the pastes with 20% sulfate content (KCSA-20CS and KCSA-20NS) than in the pastes with 5% sulfate content (KCSA-5CS and KCSA-5NS). This confirms the results observed using calorimetry, where the pastes with 20% sulfate content begin to react earlier than those with 5% sulfate content did.

**Figure 5:**
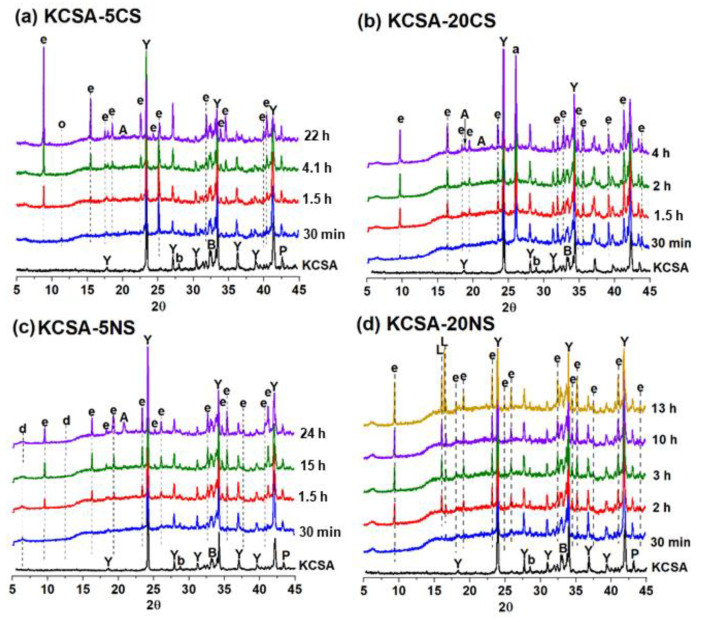
Continuous XRD diffractograms as a function of calorimetry time. Legend: Y: ye’elimite; a: anhydrite; b: belite; B: brediggite; P: periclase; A: AH_3_; e: ettringite; o: C_4_A$\bar{c}$H_11_; d: CAH_10_; L: mirabilite (Na_2_SO_4_ · 10 H_2_O).

For a clearer representation of paste hydration, Figure 5 shows the continuous XRD diffractograms selected as a function of the time at which the peaks were obtained using calorimetry (Figure 1). The formation of small ettringite peaks was observed at 30 min in all four pastes (Figure 5). In the pastes containing calcium sulfate (Figure 5a,b), the intensity of the ettringite peaks increases from 1.5 h onwards. In the KCSA-5CS paste, ettringite becomes the main product from 22 h onwards, while in the KCSA-20CS paste, in addition to ettringite being formed at 1.5 h, small peaks corresponding to AH_3_ are also observed. These peaks of AH_3_ also start to emerge after 4.1 h in the paste containing 5CS. In addition, the KCSA-5CS paste shows small peaks corresponding to calcium carboaluminate (C_4_A$\bar{c}$H_11_).

In the case of the pastes containing sodium sulfate (Figure 5c,d), the diffractograms show that ettringite becomes the main hydration product from 1.5–2 h onwards, with the peaks being most intense in the KCSA-20NS paste. The formation of peaks of AH_3_ and small peaks corresponding to calcium aluminate (CAH_10_) is also observed after 24 h in the KCSA-5NS paste. Meanwhile, in the KCSA-20NS paste, the formation of small peaks identified as corresponding to the mirabilite (Na_2_SO_4_ · 10 H_2_O) is observed from 30 min onwards, increasing in intensity with time [24].

If all the continuous diffractograms of the pastes containing calcium sulfate are analyzed, the main phase in both pastes (KCSA-5CS and KCSA-20CS) is ettringite, with the most intense peaks appearing to be those generated by the 5% sample.

In the diffractograms of the pastes containing sodium sulfate (KCSA-5NS and KCSA-20NS), the formation of ettringite as the main reaction product is also observed in the 5% and 20% samples. Both pastes also show peaks corresponding to AH_3_ that increase in intensity with an increase in the hydration time from 15 h onwards.

### 2.4. Mineralogical Characterization: Discontinuous XRD

**Figure 6:**
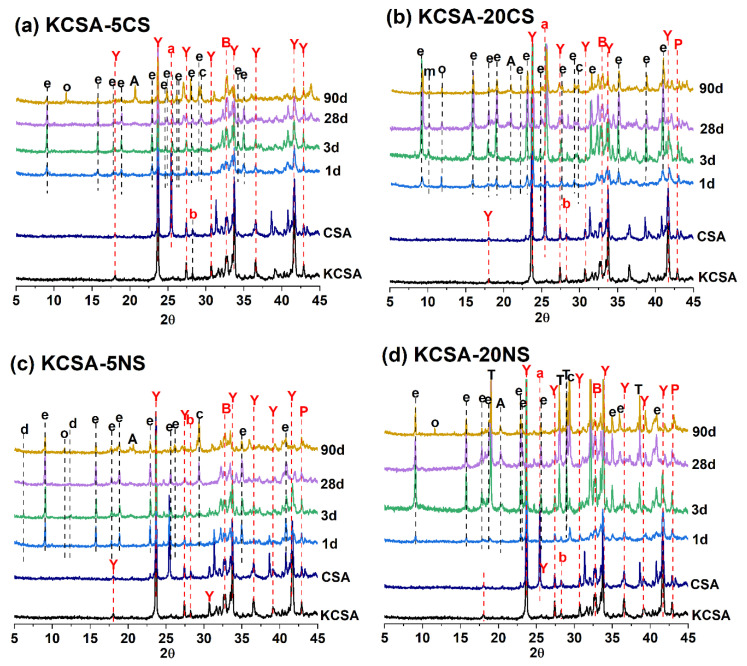
Diffractogram of the KCSA paste containing the different proportions of salts: (**a**) 5% CaSO_4_, (**b**) 20% CaSO_4_, (**c**) 5% Na_2_SO_4_, (**d**) 20% Na_2_SO_4_ at 1, 3, 28, and 90 days. Legend: **Y**: Ye’elimite (C_4_A_3_$\bar{s}$, PDF-33-0256); **b**: belite (C_2_S PDF-86-0398); **e:** ettringite (C_6_A$\bar{s}$_3_H_32_ PDF-41-1451); **A**: AH_3_ (AH_3_ PDF-74-1775); **T**: thenardite (Na_2_SO_4_ PDF-37-1465); **o**: C_4_A$\bar{c}$H_11_ (C_4_A$\bar{c}$H_11_ PDF-87-0493); **c**: calcite (CaCO_3_ PDF-86-2334); **d**: CAH_10_ (CAH_10_ PDF-12-0408); **m**: calcium monosulfoaluminate (C_4_A$\bar{s}$H_12_ PDF-83-1289); **a**: anhydrite (C$\bar{s}$ PDF-72-0916).

Figure 6 shows a portion of the diffractograms (zone spanning values 2–5Φ at 30°) for the clinker and hydrated pastes (KCSA-5CS, KCSA-20CS, KCSA-20NS, and KCSA-5NS) at 1, 3, 28, and 90 days.

In both the clinker and the calcium sulfoaluminate cement, ye’elimite (C_4_A_3_S) presents as the main phase. In addition, the cement also contains anhydrite and, in both materials, belite (C_2_S) and brediggite (Ca_7_Mg(SiO_4_)_4_), gehlenite (Al_2_Ca_2_O_7_Si), C_3_A, and periclase (MgO) present as secondary phases.

The diffractograms of the pastes containing calcium sulfate (KCSA-5CS and KCSA-20CS, Figure 6) show that as the hydration time increases, the intensity of the peaks associated with the ye’elimite and anhydrite decreases. The main hydration product, alongside AH_3_ and calcite, is ettringite. In the paste with 5% content (KCSA-5CS), calcium carboaluminate (C_4_A$\bar{c}$H_11_) also presents at 90 days. In the paste with 20% content (KCSA-20CS), the presence of calcium carboaluminate is detected after 24 h alongside small peaks attributable to calcium monosulfoaluminate.

In the case of the pastes containing sodium sulfate (Figure 6), the intensity of the ye’elimite also decreases with an increase in the hydration time. In the KCSA-5NS paste, the main product formed is ettringite, which increases in intensity up to 28 days before decreasing at 90 days, the age at which the presence of other phases such as AH_3_, C_4_A$\bar{c}$H_11_, and calcite is observed along with small peaks of CAH_10_. In the KCSA-20NS paste, the presence of C_4_A$\bar{c}$H_11_ is also observed at 90 days, while the AH_3_ and calcite are observed at 3 days and 1 day, respectively. In this paste (KCSA-20NS), CAH_10_ is not detected.

One important finding to note in the KCSA-20NS paste is that, at 1 day, the presence of Na_2_SO_4_ was not observed, nor was it observed in the continuous XRD analyses (see Figure 5). However, this phase was detected at more advanced ages, indicating that sodium sulfate dissolution followed by re-precipitation in the form of thenardite occurs. The peaks associated with the presence of thenardite increase in intensity with an increase in the hydration time. At this point, it should be noted that these pastes exhibited significant mechanical strength losses (and even cracking) between 28 and 90 days (see Figure 3).

### 2.5. Differential Thermal Analysis and Thermogravimetry (DTA/TG)

**Figure 7:**
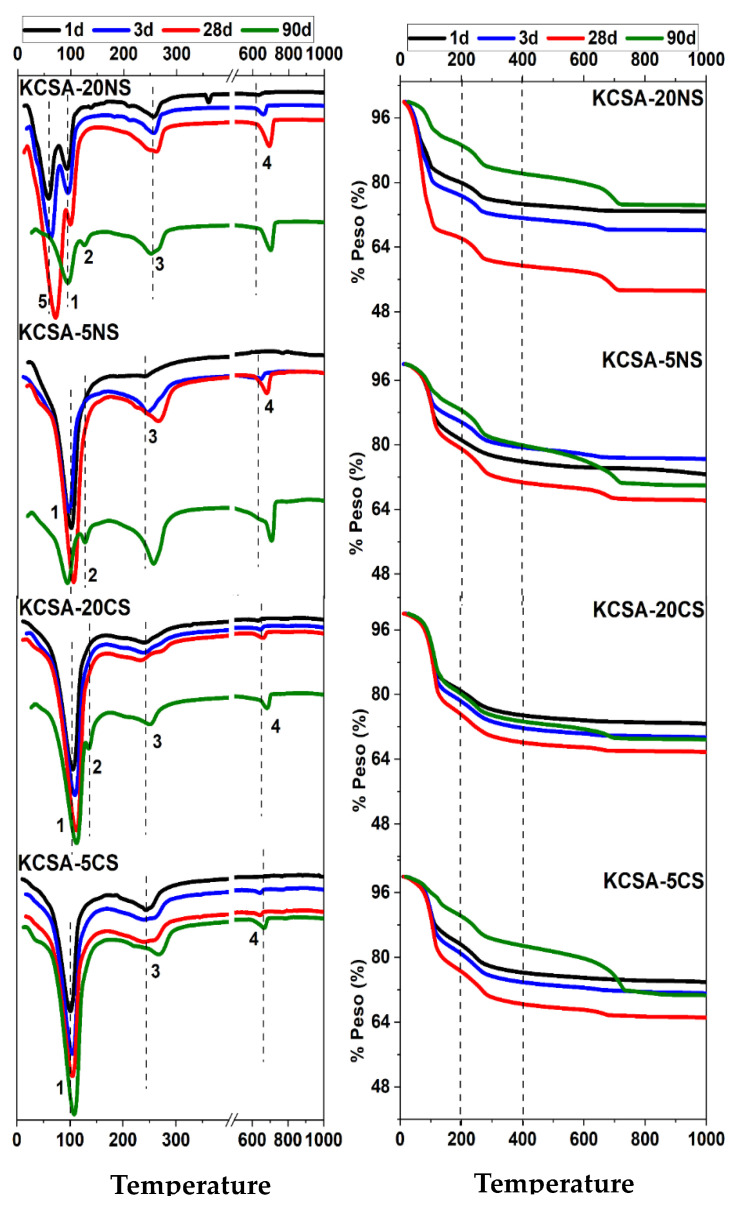
Differential thermal analysis (DTA) and thermogravimetric (TG) analysis of the KCSA pastes containing different salts.

Figure 7 presents the DTA/TG results for the hydrated KCSA pastes containing 5% and 20% CaSO_4_ and Na_2_SO_4_ at 1, 3, 28, and 90 days.

The DTA curves show the presence of a series of signals that correspond to the decomposition of the different phases mentioned above and identified using XRD. The first signal (signal 1), at around 100 °C, corresponds to the presence of ettringite. In the KCSA-20NS paste, this signal (signal 1) is detected at ~95 °C and splits, creating another signal (signal 5) at around 65 °C for the pastes at 1, 3, and 28 days. Although signal 5 is no longer detected at 90 days, another signal (signal 2) is observed at 125 °C. Signal 5 may be due to the decomposition of the calcium carboaluminate while signal 2 may be due to the decomposition of the hydrated calcium aluminate, which is not clearly observed in the KCSA-20NS paste using XRD [25].

A third signal (signal 3) then appears at close to 245 °C and shifts to 267 °C as the hydration time increases. This signal is associated with the decomposition of the aluminum hydroxide phase (AH_3_) previously observed with XRD. In the KCSA-20NS paste, at 28 days, signal 3 splits in two, standing at 247 °C and 261 °C, respectively. In the KCSA-5CS and KCSA-20CS pastes, a similar split occurs at 3 days and 28 days, respectively [23]. The signal detected at 250 °C in the KCSA-20CS paste is associated with this phase (AFm).

Finally, at values above 600 °C, the signal (signal 4) obtained corresponds to the loss of the CO_2_ in the calcium carboaluminate and the calcite, with the peak at 650 °C being attributable to the C_4_AH_11_ and the peak at around 720 °C being attributable to the calcite.

Regarding the TG curves in the 50–200 °C interval (corresponding to the phases C_4_A$\bar{s}$_3_H_32_, CAH_10_, C_4_A$\bar{c}$H_11_ and C_4_A$\bar{s}$H_12_), it can be observed how, in all the pastes, the water loss increases up to 28 days and decreases at 90 days; moreover, this water loss increases as the concentration of the salts increases. For the next interval (200–400 °C), where the AH_3_ phase is found, the weight loss increases with an increase in the hydration time and decreases with increasing CaSO_4_ concentration, while in the case of the Na_2_SO_4_ salt, the water loss is greater than 20%. The range (600–800 °C) is associated with the loss of CO_2_ from the carboaluminates and calcite.

### 2.6. Scanning Electron Microscopy (SEM/EDX)

**Figure 8:**
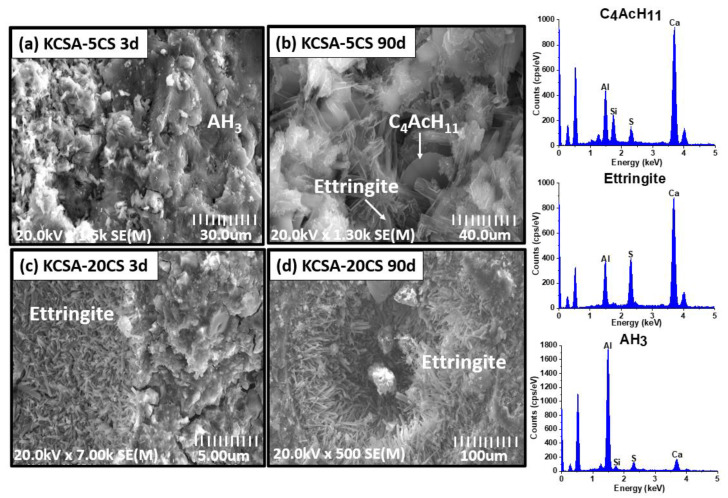
SEM micrographs of KCSA containing CaSO_4_; (**a**) KCSA-5CS at 3 days; (**b**) KCSA-5CS at 90 days; (**c**) KCSA-20CS at 3 days; (**d**) KCSA-20CS at 90 days.

**Figure 9:**
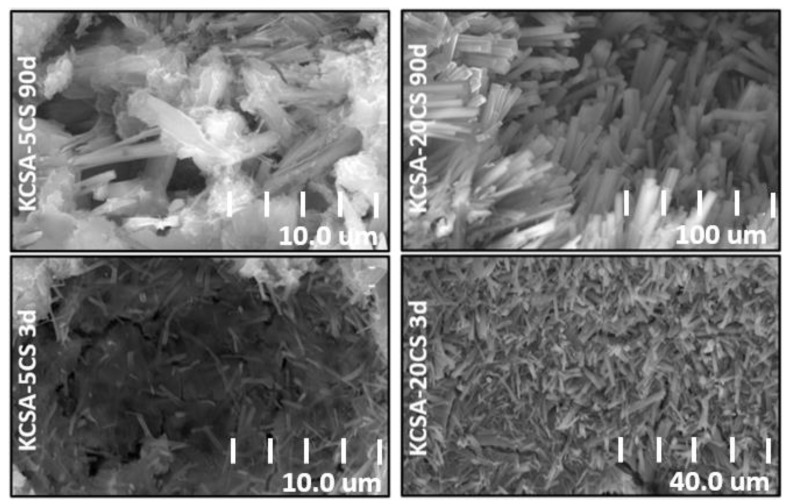
High-resolution view of the micrographs showing ettringite in the KCSA-CS pastes.

**Figure 10:**
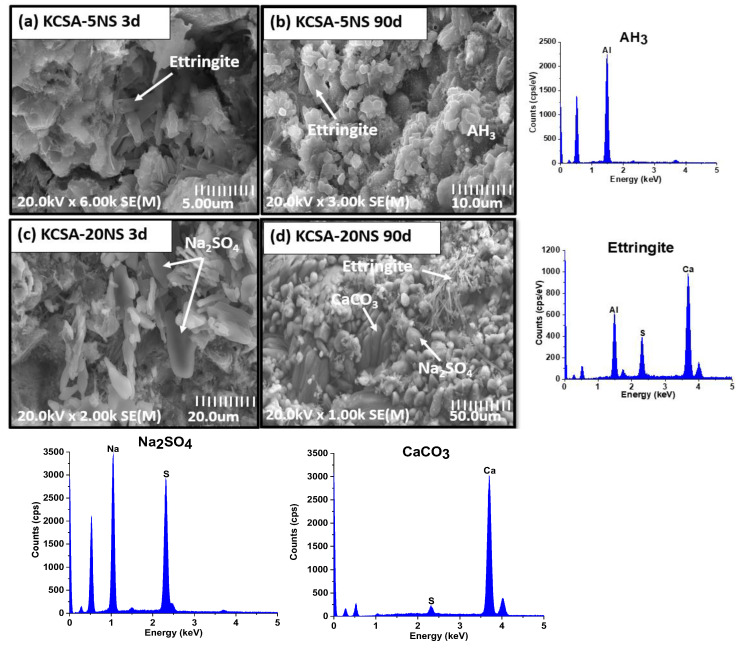
SEM micrographs of KCSA containing Na_2_SO_4_; (**a**) 5-NS at 3 days; (**b**) 5-NS at 90 days; (**c**) 20-NS at 3 days; (**d**) 20-NS at 90 days.

**Figure 11:**
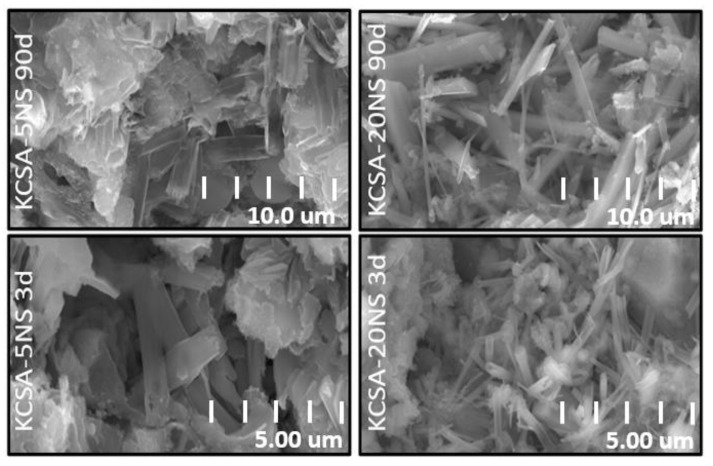
High-resolution view of the micrographs showing ettringite in the KCSA-NS pastes.

The KCSA pastes containing the different salts at 5% and 20% concentrations were analyzed at 3 and 90 days using SEM (Figure 8, Figure 9, Figure 10 and Figure 11).

Figure 8 shows four micrographs of the pastes containing calcium sulfate. In the KCSA-5CS paste, poorly crystallized AFt and AH_3_ plates are clearly observable at 3 days (Figure 8a). At 90 days, the ettringite crystals present a more defined needle or rod morphology. C_4_A$\bar{c}$H_11_ is also detected (Figure 8b). When the concentration increases to 20% (KCSA-20CS, Figure 8c,d), greater ettringite needle precipitation is observed at both 3 and 90 days. At 90 days, ettringite crystals have started to fill in the pores, helping to densify the matrix and increase the mechanical strength. As Durlo [15] indicates, the use of a 0.5 w/b ratio, which is lower than required as regards the stoichiometry, produces denser matrices and hydration products with a low degree of crystallinity, as can be seen in the micrographs in Figure 8.

Figure 9 shows the micrographs at a higher resolution than in the preceding figure. In Figure 9, the ettringite in the pastes containing calcium sulfate is clearly visible. Comparing the structure of the ettringite in the different pastes containing calcium sulfate reveals that the morphology varies slightly depending on hydration time and CaSO_4_ concentration. In the KCSA-5CS pastes, a small number of incipient ettringite needles are already visible at 3 days. When the hydration time increases to 90 days, the ettringite needles grow in size and become more abundant. When the calcium sulfate concentration is increased from 5% to 20%, the ettringite takes the form of more voluminous needles (Figure 9). This behavior was also observed by Kharchenko et al. [26]. These authors studied the variation in ettringite morphology at a constant pH of 11 while changing the CaSO_4_ concentration. They observed that a low or null CaSO_4_ concentration favors the formation of ettringite in the form of dense spheres and short, fine needles, while a higher concentration of the salt favors the formation of ettringite in the form of long, fiber-like needles that become thicker and shorter as the ions (Ca^2+^) in the starting solution increase.

SEM analysis of the pastes containing different concentrations of sodium sulfate likewise reveals ettringite needles (Figure 10a) and aluminum hydroxide plates (Figure 10b) in the paste containing 5% (KCSA-5NS). When the concentration of sodium sulfate (KCSA-20NS) increases, in addition to the above-mentioned phases, prismatic particles corresponding to thenardite (Figure 10c) are detected. At 90 days, in addition to the thenardite, calcium carbonate plates are also detected (Figure 10d). The presence at 90 days of large amounts of thenardite and calcite corroborates the XRD findings and may be responsible for the cracking and loss of cohesion of the KCSA-20NS.

Figure 11 provides a higher-resolution image of the morphology of the ettringite in the pastes (KCSA-5NS and KCSA-20NS). The figure shows that at 5% sodium sulfate content, the ettringite has a poorly defined morphology comprising dense spheres and short needles. At longer hydration times and a 20% concentration, the ettringite needles appear to be longer and slenderer. Kharchenko et al. [26] observed that at a pH of around 10, small ettringite needles form, while at pH 12 the needles become intermediate in size.

## 3. Discussion

The results obtained in this paper show that the sulfate content and type affect the kinetics, mechanical strength and nature of the reaction products formed during the hydration of the KCSA. A low sulfate content lengthens the induction period in the heat release rate curves, while high concentrations shorten it. Furthermore, while calcium sulfate has a positive impact on the development of mechanical strength, sodium sulfate exerts a less positive or even adverse impact on it, especially at advanced ages. The effect of each of these sulfates is discussed in greater detail below.

### 3.1. Hydration of the KCSA Containing Anhydrite

In terms of calorimetry (Figure 1a), both the KCSA-5CS and KCSA-20CS peak at around 1.4 h. According to Jansen [27], this peak is related to the formation of ettringite and amorphous AH_3_. The subsequent peaks are mainly associated with greater ettringite precipitation. The formation of ettringite and AH_3_ was observed using both XRD (continuous and discontinuous) and DTA/TG.

Calorimetry reveals that this first peak appears at very similar values in KCSA-H and KCSA-5CS, the peak being a little more intense in the latter. In the KCSA-20CS sample, however, the intensity is slightly lower and shifts to overlap another peak. This result suggests that in the absence of sulfate, or at low sulfate levels, a layer of reaction products is formed, essentially comprising ettringite and amorphous AH_3_. These initial reaction products form a heterogeneous surface coating that exhibits a certain degree of compaction on the clinker grains that delays the formation of secondary ettringite (longer induction period). In other words, the dissolution of the ye’elimite may be delayed because it is blocked by early hydration products like aluminum hydroxide.

With a higher calcium sulfate content (~20%), although calorimetry shows that the first peak is slightly delayed due to a combination of the lower solubility of the CaSO_4_ and dilution of the ye’elimite, after the first few minutes, the medium quickly becomes saturated with sulfate and calcium ions while the aluminum ion concentration remains relatively low compared to the pure ye’elimite clinker [8,28]. In summary, the formation of a layer of *porous* ettringite on the clinker grains is initially favored, making it easier for the reaction to occur due to diffusion thanks to the transport of water via this initial layer of porous hydrates. This would explain the shorter induction period for the KCSA-20CS pastes. At this point, it should be noted that in order to corroborate this hypothesis, it is important to carry out further studies on ion concentration in the aqueous phase during this early reaction stage.

**Figure 12:**
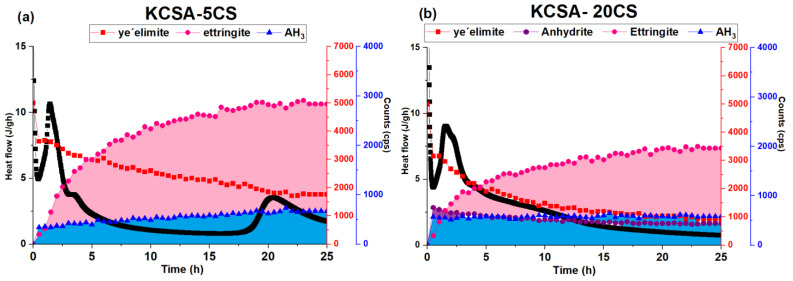
Comparison between the variation in intensity of the main peaks detected using continuous XRD and the calorimetry curves as a function of time for the pastes containing CaSO_4_ (the ye’elimite is measured on the red axis and the hydration products are measured on the blue axis).

Figure 12 correlates the calorimetry results with the continuous XRD analyses at early ages. In the KCSA-5CS paste, the peak produced at 1.4 h (Figure 12a) corresponds to the formation of a primary ettringite. The continuous XRD diffractograms obtained during the first 2 h show a slight decrease in ye’elimite and the formation of ettringite, mainly alongside amorphous AH_3_. Between the 2 and 5 h marks, calorimetry detects a small peak associated with an increase in ettringite formation. Between the 5 and 20 h marks, an induction period occurs, during which the ettringite continues to precipitate, albeit at a slower rate. The peaks detected using calorimetry at 22 and 28 h are related to the notable decrease in the intensity of the ye’elimite peak and the slight increase in ettringite and AH_3_. Since the AH_3_ is amorphous, it is difficult to identify with XRD (it should be noted that these XRD data are semiquantitative and therefore solely offer nonquantitative comparative information).

If after increasing the anhydrite concentration to 20% (KCSA-20CS) the calorimetry data are again compared with the continuous XRD analysis (Figure 12b), it becomes clear that the peaks obtained at 1.5 and 2.2 h correspond to the rapid decrease in the intensity of the ye’elimite peak and the rapid precipitation of the ettringite and AH_3_. The shoulder detected at 4 h is related to the continuing decrease in ye’elimite and the slight increase in ettringite, principally.

Mechanical strength is determined after the first 24 h (Figure 3a). The figure shows that, at 24 h, both the KCS-5CS and the KCSA-20CS pastes present higher strength values than when the clinker is hydrated with water in the absence of a calcium sulfate addition (KCSA-H). These strength values remain more or less constant in the KCSA-5CS paste while they increase substantially in the KCSA-20CS paste. In fact, the KCSA-20CS paste presents values very similar to the CSA-H commercial cement while the mechanical strengths obtained in the 5CS are similar to those of the KCSA clinker.

Several important factors influence the development of mechanical strength: (i) the degree of reaction of the material; (ii) the nature of the reaction products formed; and (iii) the porosity and pore size distribution.

***(i) Degree of reaction***. Although there are no continuous XRD data at ages greater than 24 h, ex situ XRD data are available (Figure 6). The data show that ye’elimite consumption is greatest in the 20CS paste. Ye’elimite consumption by the 5CS paste diminishes over 3–28 days, with the intensity of the ye’elimite peak decreasing more rapidly from 28 days onwards.

***(ii) Reaction products formed***. The main reaction products formed in the KCSA-5CS and KCSA-20CS pastes are ettringite and AH_3_. Theoretically, KCSA’s main hydration products include both AFt and AFm. However, AFm is only formed until all the CaSO_4_ is exhausted. In this study, no AFm was detected in the XRD patterns. Aluminum hydroxide is also a major KCSA hydration product. However, it is difficult to characterize with XRD because it mainly exists in gel form [27,29].

The results obtained by discontinuous XRD are confirmed by DTA/TG (Figure 7). The signal detected using DTA at approximately 270 °C indicates the formation of aluminum hydroxide. DTA/TG reveals signals corresponding to other phases such as CAH_10_, C_4_A$\bar{c}$H_11_, and C_4_A$\bar{s}$H_12_, which are not clearly identified by XRD.

***(iii) Porosity***. Figure 4 shows the porosity and pore distribution at 1 and 28 days for the KCSA-5CS and KCSA-20CS pastes. The total porosity decreases as the salt concentration and reaction time increase.

As stated above, these pastes’ main hydration products are ettringite and AH_3_. Morphologically speaking, ettringite is an acicular crystal with a clear direction of growth and large openings between crystals, thereby leaving more scope in the cement hydration process for pores greater than 0.1 μm. AH_3_, meanwhile, contributes to the pores measuring less than 0.01 μm. Porosity is closely related to volume reduction after hydration water consumption, while average pore diameter is related to paste homogeneity [23].

According to Li et al. [30], hydrated CSA cement pastes generally show a bimodal pore radius distribution when the CaSO_4_ does not achieve the stoichiometric molar ratio necessary for ettringite formation and a unimodal pore radius distribution when the CaSO_4_ is sufficient to achieve it [30,31]. In fact, Figure 4 shows this bimodal behavior in the KCSA-5CS paste at 1 day. As the hydration time increases, however, pore size distribution in the paste becomes unimodal. Conversely, the KCSA-20CS paste exhibits unimodal behavior at both ages. The porosity results obtained here can be interpreted as follows: until the completion of the first day of hydration, the hydration products are insufficient to fill the spaces in the cement paste. It is therefore possible to observe pores with large openings (~0.1 μm 200 nm), which represent the nonhydrated areas, and pores with smaller openings (~0.01 μm 40 nm), which principally represent the space between the hydration products. At curing times greater than 1 day, most of the nonhydrated areas are filled in by hydration products, thereby forming a unimodal pore size distribution similar to that of a hydrated CSA cement paste containing sufficient CaSO_4_ [31]. Continuous formation of hydration products fills in part of the space and densifies the material.

In short, the behavior observed in the hydration of KCSA containing anhydrite varies according to salt concentration. At 5% CaSO_4_, the mechanical strength is similar to the value obtained for the water-hydrated KCSA and the rate of hydration of the paste is slowed. Ye’elimite consumption is progressive and the primary hydration product formed is ettringite, followed by aluminum hydroxide. Increasing the anhydrite concentration to 20% results in an increase in mechanical strength and an acceleration in the paste’s hydration rate, producing similar results to those obtained for CSA cement.

3*CaO* • 3*Al*_2_*O*_3_ • *CaSO*_4_ + 2*CaSO*_4_ + 38*H*_2_*O →* 3*CaO* • *Al*_2_*O*_3_ • 3*CaSO*_4_ • 32*H*_2_*O* + 4*Al*(*OH*)_3_

### 3.2. Hydration of the KCSA Containing Na_2_SO_4_

While incorporating sulfate in the form of Na_2_SO_4_ modifies the KCSA’s hydration kinetics at early ages, its greatest effect is seen at advanced ones. The calorimetry (Figure 1b) data reveal a fairly complex heat flow rate curve with numerous peaks. The figure shows how in the KCSA-5NS, versus the KCSA-H, the 5% addition accelerates and intensifies the first peak before lengthening the induction period. The KCSA-5NS presents peaks at 15 and 25.7 h, behaving similarly to the paste containing 5% anhydrite (KCSA-5CS).

The increase in sodium sulfate concentration slightly decreases the intensity of the first peak and delays its appearance. The induction period is not seriously affected, however, and appears at similar times to those obtained in the KCSA-H paste (with no sulfate addition). As regards the sodium sulfate concentration, therefore, the initial behavior is similar to that observed with 5% and 20% calcium sulfate.

**Figure 13:**
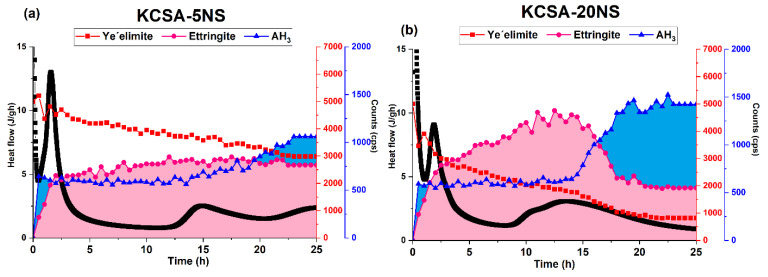
Comparison between the variation in intensity of the main peaks detected using continuous XRD and the calorimetry curves as a function of time for the pastes containing Na_2_SO_4_ (the ye’elimite is measured on the red axis and the hydration products are measured on the blue axis).

Figure 13 once again shows the intensity of the peaks of the main crystalline phases detected using continuous XRD alongside the calorimetry data in order to determine the cause of each of these peaks. Continuous XRD analysis of the same system produces the following results: during the latency period, it is possible to detect a first generation of ettringite, as small amounts of ye’elimite appear to react. By the end of the 2 h induction period, a greater amount of second-generation ettringite has formed [27].

As regards the heat of hydration, the samples with 20% content initially release more heat than the reference material, a phenomenon associated with the formation of more ettringite. The timing of the second heat flow peak follows a similar pattern in both sulfates, i.e., it appears earlier in the mixtures containing more sulfate irrespective of whether it is formed with calcium or sodium.

As the hydration time approaches 20 h, a slight drop in the intensity of the peaks associated with the ettringite is observed while the intensity of the peaks associated with the AH_3_ phase appears to increase. This behavior suggests that the last peak observed in the calorimetry curves is principally associated with the precipitation of the crystalline AH_3_ previously observed in other studies [15,17,20,32,33].

According to Zhang et al. [32] and Padilla-Encinas et al. [33], AFt and a water-rich amorphous AH_3_ are initially formed during hydration of the KCSA and CSA. The excess water in the initial amorphous AH_3_ gel is eliminated and consumed by the continuous formation of hydrated phases such as AFt, AFm, or hydrated calcium aluminates. These authors consider that the presence of a series of shoulders in the heat flow curve indicates the recrystallization of microcrystalline AH_3_ and final adjustment of the water content as the aluminum hydroxide evolves into gibbsite.

As other authors [34] indicate, the increase in pH level favors the degree of crystallinity in the AH_3_ and may even destabilize the formation of AFt. It can therefore be postulated that in these systems the presence of free sodium when the sodium sulfate reacts may increase the pH of the aqueous phase, and this increase in pH during hydration would result in the crystallization of this hydrate and the formation of bayerite [35] or gibbsite [27]. It should be borne in mind, however, that a larger crystal size could result in a more porous matrix and weaker mechanical behavior.

The results in Figure 3 show that the presence of sodium sulfate in the pastes produces mechanical strength at 1 day similar to that obtained at more advanced ages in the KCSA-H paste. Although strength increases up to 28 days, the values are lower than those obtained with water. The most important finding is the dramatic fall in mechanical strength between 28 and 90 days in the KCSA-20NS paste, which cracks completely (see Figure 3). A similar finding was made by Durlo et al. [13] when they studied the effect of 5% Na_2_SO_4_ content on the hydration of CSA cement. In that paper, they observed that at advanced ages the material cracked due to the re-precipitation of thenardite, precipitation of gypsum and carbonation of the ettringite. It could be said that internal crystal growth fractures the material.

The lower strength observed in the KCSA-5NS and KCSA-20NS pastes is very closely linked to the nature of the reaction products formed. As shown in Figure 13, consumption of the ye’elimite occurs fairly rapidly. Ettringite and AH_3_ form initially (at 3 days), explaining both the strength at this age and the intense peak in the calorimetry curve (Figure 1b). The formation of these phases at advanced ages has also been confirmed by DTA.

Thus, DTA/TG data show that at 1 day the amounts of AFt and AH_3_ increased as the sodium sulfate content rose. This trend becomes less evident as the hydration time increases. Although at 1 day the effect of the sulfate on the amounts of AFt formed is evident, this effect becomes less visible at 28 and 90 days and even diminishes. As regards AH_3_, the data show how its presence increases slightly with an increase in the hydration time.

The materials containing sodium sulfate are highly porous (see Figure 4), however, and large capillary pores (0.1–1 μm) are clearly predominant, which explains the low mechanical strength values. This greater porosity is partly associated with nonhydrated areas and partly with the cracks created by the formation of crystalline phases in the hardened paste. The crystallization (see the SEM micrographs, Figure 10 and Figure 11) causes the samples to expand, leading to cracking.

Another important aspect to highlight in the KCSA-20NS pastes is the apparent decrease in ettringite at advanced ages, favoring the formation of calcite and, above all, thenardite. The cracking of the specimens at 90 days is related to the internal stress caused by precipitation of calcite and thenardite in the matrix and the apparent decomposition of the ettringite.

The behavior observed in the KCSA-20NS pastes closely resembles that of the hydrated pastes with high alkali contents (KCSA-4M and KCSA-8M) [36]. This suggests that the high sodium sulfate content releases sulfate ions that react to form AFt, as well as releasing Na^+^ ions that may contribute to the increase in pH level. When the pH increases, the ettringite’s stability decreases, favoring carbonation [15,21]. As ettringite is unstable at high pH levels, it breaks down and releases sulfate ions, which, with the Na^+^ present in the medium, re-precipitate in the form of thenardite. SEM (Figure 10) analysis shows a high presence of thenardite and calcium carbonate (calcite) plates when the hydration time of the KCSA-20NS paste increases from 3 to 90 days. This combination of destabilization and crystallization of sulfate salts (Na_2_SO_4_) is responsible for the change in the volume of the material and its subsequent cracking [37].

As regards the pH level of the porous solution of a KCSA or CSA paste, this value is known to be around 11, clearly lower than that of a Portland cement paste (pH level of ~12.5). At this point, an additional study was conducted to determine how the pH of the KCSA-20NS paste evolved in the first 24 h. For this purpose, pastes were prepared in a 2:1 ratio and the liquid phase was extracted using a vacuum pump. This liquid phase was later used to measure the pH value [38,39]. The results obtained are shown in Table 1. It can be seen that in the first 24 h, the pH rises to values greater than 13.

The stability range of ettringite as a function of pH remains under debate. Based on studies conducted on different types of cements, several authors [15,21,36] indicate that ettringite is stable at pH levels between 9 and 13. It has been observed how, in synthetic systems and at high pH levels (>13), the morphology of the AFt changes from needle to spherical form. This behavior is very similar to that observed in the KCSA-20NS pastes (see Figure 11). At higher pH values, the ettringite becomes unstable [33].

In short, the presence of sodium sulfate affects the hydration of KCSA clinker differently depending on the salt concentration. At 5%, the mechanical strength is lower than that obtained with either the reference material (H_2_O) or calcium sulfate. In addition, the rate of hydration slows and the main hydration product is ettringite, followed by AH_3_ and thenardite. At a 20% salt content, the rate of hydration increases but the mechanical strength decreases, producing specimen fracture at 90 days due to the increased precipitation of thenardite.

## 4. Materials and Methods

### 4.1. Materials

In this study, a calcium sulfoaluminate cement clinker (KCSA) and an industrial calcium sulfoaluminate cement (CSA)—marketed by i.tech ALI PRE GREEN (supplied by Heidelberg Cement Hispania)—were used. Table 2 shows the chemical composition determined using X-ray fluorescence and the mineralogical composition obtained using X-ray diffraction, including quantitative analysis using the Rietveld method in the Topas application.

Figure S2 shows the granulometric analyses of the calcium sulfoaluminate clinker (KCSA) and the calcium sulfoaluminate cement (CSA). These laser diffraction analyses were performed using a COULTER LS 130 granulometric analyzer with a measurement range of 0.1–900.0 μm. In total, 90% of the particles in the KCSA were smaller than 35 μm; in the CSA, 90% of the particles were smaller than 11.51 μm. The clinker had a density of 2.83 g/mL and the cement had a density of 2.90 g/mL. The Blaine finenesses were 408.82 m^2^/kg and 474.6 m^2^/kg for the KCSA and CSA, respectively.

### 4.2. Procedure

To the KCSA, 5 and 20% Na_2_SO_4_ and CaSO_4_ were incorporated via solid substitution. To determine the evolution of the mechanical strength, pastes were prepared from solid mixtures of KCSA with the abovementioned salts, with a constant water/binder ratio of 0.5. A prismatic mold (6 test specimens of 1 × 1 × 6 cm^3^) was half-filled and 60 strokes were given on the shaking table so that the paste was distributed homogeneously and no pores were left in the test specimen. The mold was then filled and the 60 strokes were repeated. Once the molds were filled, the paste was cured in a climatic chamber for 24 h at 21 °C with a 95% relative humidity. The test specimens were then demolded, labelled and stored again in the climatic chamber until the test age (1, 3, 28 and 90 days). At the selected age, the specimens were broken using compression. The equipment used for this was an Ibertest press (Autotest-200/10-SW).

After breaking the specimens, part of the material obtained was ground in an agate mortar and mixed with isopropanol to halt the hydration process. The mixture was then shaken for 3 min and filtered under vacuum. Next, to remove the leftover isopropanol, the mixture was dried in a vacuum desiccator for 3 days, after which it was characterized using XRD, DTA/TG, and MRI. Some of the unground pieces were immersed in a jar of isopropanol for 7 days for later use in SEM microstructural analysis and mercury intrusion porosimetry.

The porosimetry equipment used was a MICROMERITICS Autopore IV 9500 capable of reaching pressures of up to 3200 Psi, the equivalent of a pore size of 0.0067 μm.

The continuous XRD equipment used was a BRUKER D8 ADVANCE made up of a 3 kW high-voltage generator, an X-ray tube with a copper anode (Cu Kα_1,2_ radiation, 1.540 Å) that usually operates at 40 kV and 50 mA, and a Lynxeye detector with a 3 mm antiscatter slit and a Ni K-beta (0.5%) filter without a monochromator (does not remove Kα_2_). The KCSA and salt mixture was shaken for 3 min and the sample holder was filled and covered with Kapton. Sample readings were taken every 30 min in a 5–45° range at a time/step of 0.9 s with a step size of 0.01973°.

Discontinuous XRD analysis was performed using the same equipment as for continuous XRD. Analysis was performed using a 6 mm variable divergence slit in a 5–60° 2θ range at a time/step of 0.5 s with a step size of 0.02°.

DTA/TG was performed using a TA SDT Q 600. The recording conditions were as follows: heating from room temperature to 1000 °C at a rate of 10 °C/min in a platinum crucible under a nitrogen atmosphere. The measurement sensitivity was 0.001 °C for DTA and 0.1 μg for TG.

Scanning electron microscopy and energy-dispersive X-ray (SEM/EDX) were performed using a HITACHI S-4800 microscope. The scanning microscope employed a field emission gun (FEG) with a resolution of 1.4 nm. This device was equipped with a backscattered scanning electron microscope (BSEM), a BRUKER X-ray detector, QUANTAX 400 microanalysis software and five monitored axes. The samples were vacuum-dried and metallized with charcoal.

The heat release rate and the total heat associated with the hydration reactions were also analyzed using isothermal conduction calorimetry, employing a THERMOMETRIC TAM AIR device operating at 25 °C. To this end, pastes with the same 0.5 water/binder ratio were prepared. Measures of 10 g of the cementing mixture and 5 g of water were mixed ex situ for 3 min. A 7.5 g sample of this mixture was inserted in the calorimeter. For the reference material, distilled water was used.

## 5. Conclusions

This paper analyzes the effect of alkaline salts (Na_2_SO_4_) and alkali earth (CaSO_4_) on the hydration of commercial calcium sulfoaluminate clinker (KCSA) at 1, 3, 28, and 90 days. The results are compared with the reference clinker and reference calcium sulfoaluminate cement, both hydrated with water. The general conclusions that can be drawn from this paper are summarized below.

The sulfate salt type (CaSO_4_ or Na_2_SO_4_) and quantity (5% or 20%) affect the hydration kinetics, type of reaction products formed, and development of mechanical strength.

Low sulfate concentrations (~5%) accelerate primary AFt formation but slow secondary AFt formation. However, higher quantities (20%) slightly delay the formation of primary AFt and accelerate the formation of secondary AFt. These phenomena are complex and require more detailed study. Nevertheless, it is believed that they may be related to the type and morphology of the AFt initially formed.

Hydration of the KCSA in the presence of calcium sulfate leads to the formation of ettringite as the main hydration product, along with other phases such as C_4_A$\bar{c}$H_11_, AH_3_, CAH_10_, and calcite. Hydration of KCSA containing sodium sulfate also induces the formation of ettringite, alongside other products such as AH_3_ and calcite. Increasing the hydration time as a function of added Na_2_SO_4_ content causes part of the AFt to break down to form thenardite and calcite.

The incorporation of calcium sulfate salts has a positive effect on the development of mechanical strength in KCSA. Adding sodium sulfate, however, has less positive or even adverse effects, especially at advanced ages. This behavior is largely associated with the fact that the presence of Na_2_SO_4_ increases the pH value, which partly destabilizes the AFt formed, favoring carbonation and the precipitation of thenardite, which can cause the specimens to crack and break.

## Figures and Tables

**Table 1:** pH values of the aqueous phase of the KCSA-20NS paste.

	pH MEASUREMENTS					
	30 min	1 h	2 h	5 h	7 h	24 h
KCSA-20NS	11.39	11.40	11.64	12.42	12.51	13.19

**Table 2:** Chemical and mineralogical analysis of the KCSA clinker.

Chemical Composition			Mineralogical Composition		
Oxides	KCSA	CSA	^2^ ICDD PDF	KCSA	CSA
CaO	40.82%	41.50%	Ye’elimite, C_4_A_3_s, (PDF = 33-0256)	68.4 ± 0.9%	52.6 ± 0.4%
SiO_2_	9.51%	8.14%	Brediggite, Ba_0.293_Ca_13.467_Mg_1.81_Mn_0.43_O_32_Si_8_ (PDF = 36-399)	7.40 ± 0.4%	5.0 ± 0.5%
Al_2_O_3_	29.37%	23.20%	Belite, C_2_S, (PDF = 86-0398)	16.90 ± 1.0%	14.9 ± 0.4%
Fe_2_O_3_	1.32%	1.05%	Periclase, MgO, (PDF = 4-829)	3.67 ± 0.2%	2.8 ± 0.3%
MgO	4.13%	3.22%	Gehlenite, Al_2_Ca_2_O_7_Si, (PDF = 35-755)	1.90 ± 0.4%	1.6 ± 0.5%
Na_2_O	1.06%	0.86%	C_3_A, (PDF = 70-0839)	1.75 ± 0.4%	1.0 ± 0.2%
K_2_O	0.47%	0.44%	Anhydrite, CaSO_4_ (PDF = 72-0916)		22.1 ± 0.4%
TiO_2_	0.39%	0.32%			
SO_3_	9.92%	18.36%			
Others	1.70%	1.35%			
^1^ LOI	1.31%	1.45%	Crystallography Open Database		

^1^ Loss on ignition; ^2^ International Centre for Diffraction Data.

## Data Availability

Data are contained within the article and supplementary materials.

## Supplementary Materials

The following supporting information can be downloaded at: https://www.mdpi.com/article/10.3390/molecules28227651/s1, Figure S1: Continuous XRD diffractograms for the KCSA pastes with each of the salts; Figure S2: KCSA particle size.
