# Supplementary material for: Effect of Na versus Ca Sulfate Salts on the Hydration of Calcium Sulfoaluminate Clinker
Source: Molecules. 2023 Nov 18;28(22):7651. doi: 10.3390/molecules28227651 (PMC10674699; doi:10.3390/molecules28227651)
Supplement: Supplementary file 1 [file molecules-28-07651-s001.zip › molecules-2677192-supplementary.pdf]

molecules

MDPI

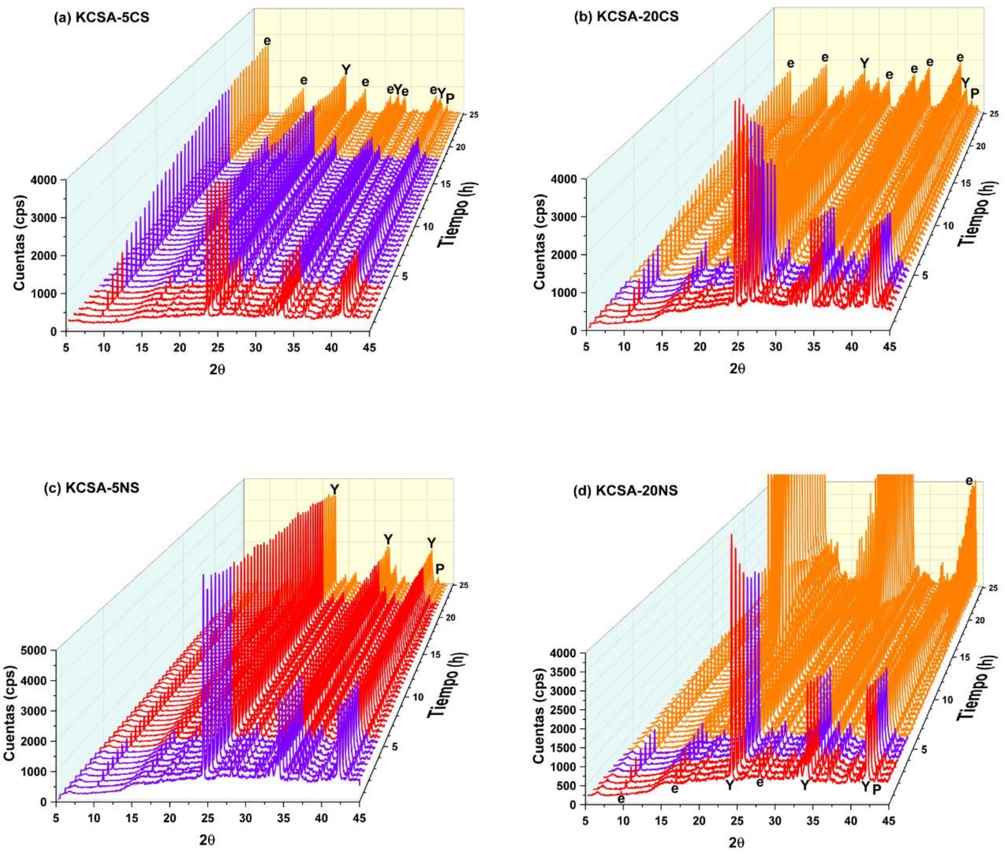

Figure S1. Continuous XRD diffractograms for the KCSA pastes with each of the salts.

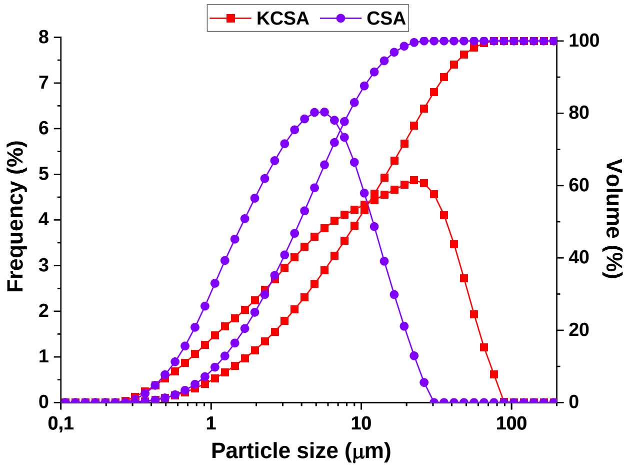

Figure S2. KCSA particle size.
